# The Burden of Epilepsy in Thailand: Estimating Societal Costs and Quality of Life

**DOI:** 10.1155/bn/2885542

**Published:** 2026-03-05

**Authors:** Prapassara Sirikarn, Surachai Phimha, Luxzup Wattanasukchai, Sineenard Mungmanitmongkol, Somsak Tiamkao

**Affiliations:** ^1^ Department of Epidemiology and Biostatistics, Faculty of Public Health, Khon Kaen University, Khon Kaen, Thailand, kku.ac.th; ^2^ Integrated Epilepsy Research Group, Khon Kaen University, Khon Kaen, Thailand, kku.ac.th; ^3^ Department of Public Health Administration, Health Promotion, and Nutrition, Faculty of Public Health, Khon Kaen University, Khon Kaen, Thailand, kku.ac.th; ^4^ Clinical Epidemiology Unit, Faculty of Medicine, Khon Kaen University, Khon Kaen, Thailand, kku.ac.th; ^5^ Division of Nursing, Srinagarind Hospital, Faculty of Medicine, Khon Kaen University, Khon Kaen, Thailand, kku.ac.th; ^6^ Division of Neurology, Department of Medicine, Faculty of Medicine, Khon Kaen University, Khon Kaen, Thailand, kku.ac.th

**Keywords:** carer, direct costs, economic, indirect costs, quality of life, seizure

## Abstract

**Background:**

Epilepsy imposes both economic and quality‐of‐life (QOL) burdens. Therefore, this study is aimed at evaluating the costs of illness and QOL in patients with epilepsy (PWE) and their caregivers and to examine the differences in costs and QOL across patient characteristics.

**Methods:**

This prevalence‐based cost‐of‐illness study was conducted from a societal perspective. Data were collected from patients who attended the Epilepsy Clinic at Srinagarind Hospital, Khon Kaen University, between April and August 2023. QOLs were assessed using the Quality of Life in Epilepsy Inventory‐31 (QOLIE‐31) for PWE and the EuroQoL‐5D‐5L (EQ‐5D‐5L) for caregivers. A generalized linear model was used to assess the differences in costs and QOL across patient and caregiver characteristics.

**Results:**

A total of 214 subjects were enrolled in this study, comprising 129 PWE and 85 caregivers. The mean total cost of illness per outpatient visit was 452.27 PPP‐USD, with 58.0% attributed to direct medical costs, 24.4% to direct nonmedical costs, and 17.6% to indirect costs. Direct medical costs differed significantly based on employment status (p value = 0.031) and seizure control (p value = 0.018). Direct nonmedical costs showed differences by residential distance (p value < 0.001). Indirect costs also showed differences by age (p value = 0.019) and residential distance (p value = 0.002). The lifetime productivity loss from unemployment reached 14638.37 PPP‐USD per patient. The mean overall QOLIE‐31 score was 74.04 points, and the mean EQ‐5D‐5L index was 0.90. There were differences in QOL based on age and seizure control in PWE, as well as age and unemployment status in caregivers.

**Conclusion:**

Direct medical costs were the largest component of total costs. Cost differences were found across age, employment status, residential distance, and seizure control. QOL varied by age and seizure control in PWE and by age and unemployment status in caregivers.

## 1. Introduction

Epilepsy is an emergency brain disorder that can affect comorbidities, disabilities [1, 2], and quality of life (QOL) of both patients and their caregivers [3–5]. A previous systematic review and meta‐analysis showed that there were 45.9 million patients with epilepsy (PWE) (95% confidence interval [CI]: 39.3–54.6) globally, and among these, there were 126055 epilepsy‐related deaths and 13.5 million disability‐adjusted life‐years (DALYs) [6].

As a chronic neurological disease, epilepsy requires continuous treatment and monitoring to prevent recurrence and can lead to physical and mental disabilities [7]. Prolonged treatment imposes substantial economic burdens on patients, families, and the healthcare system. A recent study revealed that the average annual cost for PWE in low‐income countries was 55 USD, whereas in high‐income countries, it was 4476 USD in 2019. In addition, there was a mean direct cost of 1687 USD and an indirect cost of 2780 USD per person [8]. Similar to other common chronic diseases, the significant cost burden of epilepsy is primarily generated by indirect costs, which account for over 50% of the total costs [9–12]. Furthermore, direct nonmedical costs were the dominant cost for PWE [13, 14], which covered a significant portion of patients′ out‐of‐pocket payments. Direct medical costs are also substantial due to the necessity for ongoing medication, emergency care, and hospitalizations [15, 16]. The magnitude of all these costs varies considerably depending on the characteristics and health‐related conditions of PWE [11, 17].

Despite the clear global economic burden, reliable cost data are highly variable across different socioeconomic settings. Economic evidence in Southeast Asia reported mean direct costs were 0.32 USD in Malaysia [18], 1043.45 PPP‐USD in Thailand [19], and 8148 USD for Thai patients aged ≤ 25 years with drug‐resistant focal epilepsy who were surgical candidates [20]. However, these studies were limited to direct costs, and comprehensive assessments of indirect costs in Southeast Asia have not been adequately reported.

In addition to the economic burden that PWE endure, protracted treatment and seizures can have adverse effects on their social and mental well‐being. For example, PWE may experience anxiety, depression, and social isolation. Additionally, certain PWE resign from their jobs and lose earnings, which is related to a high risk of depression [21] and a worse QOL [22, 23]. Moreover, these negative impacts can also affect caregivers [4, 24].

Current literature demonstrates significant omissions pertaining to the economic and social burdens of epilepsy in Thailand. Specifically, a comprehensive evaluation encompassing all cost components (direct and indirect), alongside a detailed assessment of the QOL for both PWE and their caregivers, is critically needed to inform policy and resource allocation.

Therefore, this study is aimed at investigating the epilepsy‐related direct and indirect costs and the QOL of PWE and their caregivers, and to compare these outcomes according to patient sociodemographic and clinical characteristics.

## 2. Materials and Methods

### 2.1. Design and Setting

This prevalence‐based cost‐of‐illness study was performed from a societal perspective and conducted among PWE and their caregivers who attended the Epilepsy Clinic at Srinagarind Hospital, a major tertiary referral center affiliated with Khon Kaen University between April and August 2023.

### 2.2. Participant Selection and Recruitment

All PWE and their caregivers who visited the Epilepsy Clinic at Srinagarind Hospital, Khon Kaen University, from April to August 2023 and fulfilled the eligibility criteria were required to provide informed consent.

#### 2.2.1. The Criteria for PWE

Participants were adults (≥ 18 years) diagnosed with epilepsy who had received treatment at Srinagarind Hospital for at least 1 year and attended outpatient clinic visits in person.

#### 2.2.2. The Criteria for Caregivers

Caregivers were adults (≥ 18 years) who accompanied the PWE to clinic visits and provided direct, unpaid care.

### 2.3. Data Collection

Data were collected in four parts: characteristics, direct costs, indirect costs, and QOLs.

Direct costs included both medical and nonmedical costs. Medical costs included expenditures for epilepsy‐related medication, laboratory tests, rehabilitation, and additional healthcare services extracted from the hospital information system (HIS), while direct nonmedical costs included the costs of transportation, meals, and accommodation. The questionnaire collected the characteristics and indirect costs that the participants were required to complete.

The QOL of PWE was assessed using the Thai version of the Quality of Life in Epilepsy Inventory‐31 (QOLIE‐31), which consists of 31 items across seven domains. The QOLIE‐31 overall score was calculated using the weighted average of the multi‐item scale scores ranging from 0 to 100 points, with 0 indicating the worst QOL and 100 indicating the best QOL. Individuals with *T*‐scores below 50 points were identified as having a QOL lower than the mean for the epilepsy cohort [25, 26] and were consequently categorized as having a low QOL. The assessment of caregiver QOL utilized the Thai version of EuroQoL‐5D‐5L (EQ‐5D‐5L), which comprises five dimensions, each containing five levels (no problems, slight problems, moderate problems, severe problems, and extreme problems), as well as a visual analogue scale (VAS) ranging from 0 to 100 points [27]. Responses in each dimension indicating no problems were classified as no problems, whereas the other responses were classified as problems. Index scores were calculated based on the level of impairment across the five dimensions. These scores range between less than 0 and 1 point, with 1 representing unimpaired health, 0 denoting health status as worse or equivalent to death, and below 0 indicating a health status worse than death [28].

### 2.4. Cost Calculation

This study evaluated both direct and indirect costs from a societal perspective. All costs were converted into 2023 purchasing power parity in US dollars (PPP‐USD) using the purchase power parity conversion rate calculated by the International Monetary Fund (IMF) (11.463 THB = 1 PPP − USD) [29].

Direct medical costs encompassed all epilepsy‐related costs incurred per outpatient visit and were recorded in the HIS. Direct nonmedical and indirect costs were calculated based on participant‐reported data. Total direct nonmedical costs covered transportation, meals, and accommodation. Indirect costs were categorized into three components reflecting different time horizons: (1) productivity loss due to missed workdays for PWE and their family caregivers associated with clinical attendance (calculated per visit), (2) productivity loss from seizure‐related absenteeism in the past month (calculated monthly), and (3) lifetime productivity loss attributable to epilepsy‐related unemployment. For each cost category, patients who did not incur the respective costs were assigned zero PPP‐USD.

The equations for calculating the indirect costs are as follows:

1. Productivity loss due to missed workdays for attending appointments = (patient′s daily wage rate x number of missed workdays) + (caregiver′s daily wage rate *x* number of missed workdays).
2. Productivity loss from missed workdays due to seizure recurrence in the past month = (patient′s daily wage rate *x* number of missed workdays) + (caregiver′s daily wage rate *x* number of missed workdays).
3. Productivity loss due to unemployment caused by epilepsy was calculated using the human capital approach to estimate lifetime productivity losses based on each patient′s preunemployment monthly salary, occupation, and year of job loss. Historical income values from the year of job loss to 2023 were adjusted to 2023 constant prices using annual consumer price inflation rates from the IMF [30]. Future productivity losses (2024 to retirement age 60) were maintained at 2023 constant prices and discounted to present value using a 3% annual discount rate [31]. The total lifetime productivity loss was calculated as:

Lost productivity=past losses:job loss year to 2023+future losses:2024 to retirement.

### 2.5. Statistical Analysis

The characteristics of PWE and caregivers were reported as frequency and percentage for categorical data and mean with standard deviation (SD) for continuous data.

Cost data were reported as means with SD alongside medians with interquartile ranges (IQR) and ranges (minimum–maximum), consistent with health economic evaluation guidelines [32, 33]. The 95% CIs for mean costs were estimated using bootstrap methods with 1000 iterations to account for skewed distributions.

QOL of PWE and caregivers was measured using the mean with SD and the percentage of low QOL with 95% CI. For PWE, low QOL was defined as *T*‐scores below 50. For caregivers, QOL was classified as no problems and any problems (including slight problems, moderate problems, severe problems, and extreme problems).

To characterize the distribution of costs and QOL across socioeconomic status, geographical location, and disease severity [34], we employed univariate generalized linear models (GLMs) to compare costs and QOL by patient characteristics. For cost outcomes, GLMs with gamma distribution and log‐link function were used to assess differences in costs, with results reported as ratios of mean costs with 95% CI. For QOL outcomes, GLMs with binomial distribution and identity‐link function with robust variance were used to estimate differences in low QOL, with results reported as percentage differences with 95% CI. To account for all domain‐specific multiple comparisons in QOL outcomes for both patients and caregivers, *p* values were adjusted using the Benjamini–Hochberg procedure to control the false discovery rate (FDR) [35]. All statistical analyses were performed using STATA 18.5.

### 2.6. Sensitivity Analysis

We performed sensitivity analyses to test the robustness of our productivity loss estimates by varying assumptions about income recovery following job loss. Four scenarios were examined, representing income loss ranging from 25% to 100%. In the base case, we assumed complete income loss (100%), representing patients unable to return to work and without adequate disability support. We then examined three progressively more conservative scenarios. Under 75% income loss, patients were assumed to recover 25% of their previous income through part‐time work, informal employment, or limited disability benefits. Under 50% income loss, patients were assumed to recover half of their previous income through substantial reemployment or disability support. The most conservative scenario (25% income loss) assumed patients recovered 75% of their previous income, representing minimal long‐term productivity impact. All scenarios maintained the same discount rate (3%) and inflation adjustment methods.

## 3. Results

### 3.1. Characteristics of Participants

During the study period from April to August 2023, 256 PWE attended the epilepsy clinic. A total of 129 PWE met the eligibility criteria and agreed to participate (participation rate 50.4%). Additionally, 85 caregivers were enrolled. The majority of PWE were female (59.7%). The mean age of PWE was 44.20 years old (SD = 17.94). Among these patients, 85 PWE (65.9%) arrived at the epilepsy clinic accompanied by caregivers. Of these caregivers, 36.5% were parents of the PWE (Table 1).

**Table 1:** Characteristics of PWE and caregivers.

Characteristics	*n*	(%)
**Epilepsy patients (** **n** = 129 **)**		
Sex		
Male	52	(40.3%)
Female	77	(59.7%)
Age (years)		
< 20	8	(6.2%)
20–29	26	(20.2%)
30–39	28	(21.7%)
40–49	17	(13.2%)
50–59	18	(13.9%)
≥ 60	32	(24.8%)
Mean (SD)	44.20	(17.94)
Median (min:max)	40.91	(18.26:83.26)
Level of education		
None	1	(0.8%)
Primary school	18	(13.9%)
Secondary school	34	(26.4%)
Vocational and technical education	18	(13.9%)
Undergraduate	49	(38.0%)
Graduate	9	(7.0%)
Primary occupation		
None/retirement	48	(37.2%)
Agriculturalist/merchant	32	(24.8%)
Government official	20	(15.5%)
State enterprise employee	6	(4.6%)
Private sector employee	6	(4.6%)
Student	6	(4.6%)
Monk	2	(1.6%)
Others	9	(7.0%)
Number of seizures in last 4 weeks		
Mean (SD)	1.91	(5.06)
Median (min:max)	0	(0:31)
Caregivers (*n* = 85)		
Sex		
Male	23	(27.1%)
Female	62	(72.9%)
Age (years)		
< 30	7	(8.2%)
30–39	10	(11.8%)
40–49	23	(27.1%)
50–59	23	(27.1%)
≥ 60	22	(25.9%)
Mean (SD)	51.03	(13.49)
Median (min:max)	50.97	(22.87:80.49)
Level of education		
Primary school	19	(22.4%)
Secondary school	16	(18.8%)
Vocational and technical education	8	(9.4%)
Undergraduate	27	(31.8%)
Graduate	15	(17.6%)
Primary occupation		
None/retirement	20	(23.5%)
Agriculturalist/merchant	27	(31.8%)
Government official	22	(25.9%)
Student	1	(1.2%)
Others	15	(17.6%)
Patient‐caregiver relationship		
Parents	31	(36.5%)
Partner	30	(35.3%)
Son/daughter	10	(11.8%)
Relative	9	(10.6%)
Others	5	(5.9%)
Time spent caring for patients (hours/week)		
0–24	19	(22.4%)
25–48	3	(3.5%)
49–72	7	(8.2%)
73–96	11	(12.9%)
97–120	6	(7.1%)
121–144	4	(4.7%)
145–168	35	(41.2%)
Mean (SD)	101.73	(64.02)
Median (min:max)	112	(0:168)

### 3.2. Cost of Illness

The total cost per outpatient visit was equal to 452.27 PPP‐USD (95% CI: 341.68–649.56), delineated into 58.0% for direct medical costs (262.04 PPP‐USD), 24.4% for direct nonmedical costs (110.43 PPP‐USD), and 17.6% for indirect costs (79.80 PPP‐USD). The most significant portion of direct nonmedical expenses was transportation, which accounted for 65.0% of direct nonmedical costs (Table 2). Additional indirect costs beyond clinic visits included mean monthly productivity loss from seizure‐related absenteeism of 23.80 PPP‐USD and mean lifetime productivity loss from unemployment of 14638.37 PPP‐USD (Table 3).

**Table 2:** Cost of illness per outpatient visit in PWE (*n* = 129).

Costs (PPP‐USD)	Mean	SD	Median	IQR	Min	Max	%
1. Direct medical costs							
1.1 Medication	257.68	823.23	109.92	43.97, 238.16	0.00	9159.91	57.0%
1.2 Nursing services	4.36	0.00	4.36	4.36, 4.36	4.36	4.36	1.0%
Total direct medical costs	**262.04**	**823.23**	**114.28**	**48.33, 242.52**	**4.36**	**9164.27**	**58.0%**
95% CI: Lower	**167.43**		**86.80**				
Upper	**456.87**		**136.61**				
2. Direct nonmedical costs							
2.1 Transportation	71.77	73.18	43.62	26.17, 87.24	0.00	436.19	15.9%
2.2 Meals	33.58	44.15	21.81	8.72, 46.62	0.00	261.71	7.4%
2.3 Accommodation	5.08	23.47	0.00	0.00, 0.00	0.00	191.92	1.1%
Total direct nonmedical costs	**110.43**	**117.27**	**69.79**	**43.62, 130.86**	**0.00**	**776.41**	**24.4%**
95% CI: Lower	**93.55**		**61.07**				
Upper	**132.06**		**95.96**				
3. Indirect costs							
Lost productivity due to missed workdays caused by a hospital visit							
PWE	24.89	116.49	0.00	0.00, 0.00	0.00	872.37	5.5%
Caregivers	54.91	130.80	0.00	0.00, 43.62	0.00	872.37	12.1%
Total indirect costs	**79.80**	**200.83**	**0.00**	**0.00, 69.79**	**0.00**	**1395.80**	**17.6%**
95% CI: Lower	**50.69**		**0.00**				
Upper	**120.75**		**26.17**				
Total costs	**452.27**	**862.49**	**275.93**	**144.73, 497.03**	**39.26**	**9251.51**	**100.0%**
95% CI: Lower	**341.68**		**218.80**				
Upper	**649.56**		**310.26**				

*Note:* The bolded values represent the total costs for each section, including: (1) direct medical costs, (2) direct nonmedical costs, (3) indirect costs, and (4) total costs.

**Table 3:** Additional indirect costs beyond clinic visit attendance in PWE (*n* = 129).

Costs (PPP‐USD)	Mean	SD	Median	IQR	Min	Max
1. Lost productivity from missed workdays due to suffering a recurrence of seizures within a 1‐month period (per month)						
PWE	20.69	99.47	0.00	0.00, 0.00	0.00	872.37
Caregivers	3.11	17.37	0.00	0.00, 0.00	0.00	174.47
Total	23.80	100.33	0.00	0.00, 0.00	0.00	872.37
95% CI: Lower	10.31		0.00			
Upper	45.68		0.00			
2. Lost productivity due to unemployment caused by epilepsy (lifetime costs)	14638.37	61200.51	0.00	0.00, 0.00	0.00	359217.20
95% CI: Lower	5169.17		0.00			
Upper	26455.61		0.00			

To assess the robustness of this lifetime productivity loss estimate, a sensitivity analysis was performed under varying income loss assumptions. When the assumed income loss was reduced to 75%, 50%, and 25%, the mean estimated lifetime productivity loss ranged from 3659.59 PPP‐USD to 10978.78 PPP‐USD (Supporting Information 1: Table S1).

The indirect costs in elderly PWE differed significantly from those in adults, with reductions of 66.6%. PWE with employment observed a 57.9% decline in direct medical costs. Moreover, PWE with controlled seizures (no seizure in the last month) experienced a 60.3% decrease in direct medical costs compared with those with uncontrolled seizures, whereas PWE residing outside the province of the hospital led to an increase in direct nonmedical costs by 245.6%, indirect costs by 234.4%, and total costs of illness by 118.5% (Table 4).

**Table 4:** The difference in costs of illness (*n* = 129).

Factors	n	Mean (PPP‐USD)			
		Direct medical costs	Direct nonmedical costs	Indirect cost	Total costs
Sex					
Male	52	203.42	88.81	90.51	382.74
Female	77	301.63	125.03	72.57	499.23
Mean costs ratio		1.483	1.408	0.801	1.304
95% CI		0.554–3.967	0.961–2.063	0.337–1.908	0.695–2.449
p‐value		0.433	0.079	0.617	0.409
Age (years)					
< 60	97	297.69	120.48	95.60	513.77
≥ 60	32	153.98	79.99	31.90	265.87
Mean costs ratio		0.517	0.664	0.334	0.517
95% CI		0.166–1.608	0.440–1.002	0.133–0.837	0.260–1.028
p‐value		0.255	0.051	0.019	0.060
Level of education					
Basic	71	312.19	96.80	107.20	516.19
Post‐secondary	58	200.65	127.13	46.25	374.03
Mean costs ratio		0.643	1.313	0.431	0.725
95% CI		0.250 to 1.652	0.917 to 1.881	0.162 to 1.149	0.397 to 1.323
*p* value		0.359	0.137	0.092	0.294
Employment status					
Unemployed	58	384.52	98.91	61.07	544.50
Employed	71	161.99	119.85	95.10	376.94
Mean costs ratio		0.421	1.212	1.557	0.692
95% CI		0.192–0.925	0.848–1.731	0.666–3.643	0.386–1.242
*p* value		0.031	0.291	0.307	0.217
Residential distance					
Inside province	51	186.03	44.44	33.01	263.49
Outside province	78	311.74	153.58	110.39	575.71
Mean costs ratio		1.676	3.456	3.344	2.185
95% CI		0.644–4.363	2.549–4.686	1.579–7.082	1.256–3.801
*p* value		0.290	< 0.001	0.002	0.006
Seizure‐free					
No	52	409.49	110.89	68.36	588.74
Yes	77	162.47	110.12	87.52	360.11
Mean costs ratio		0.397	0.993	1.280	0.612
95% CI		0.185–0.851	0.682–1.445	0.541–3.032	0.348–1.077
*p* value		0.018	0.971	0.574	0.088

### 3.3. QOL of PWE

The average overall QOLIE‐31 was 74.04 points (95% CI: 71.04–77.04). A total of 23% of PWE had a low QOL (95% CI: 16.0%–30.5%). The Overall QOL subscale had the highest percentage of low QOL (30.2%), whereas the energy or fatigue dimension had the lowest percentage (18.6%) (Table 5).

**Table 5:** Quality of life in PWE (*n* = 129).

Domains	Possible score	Mean	(SD)	Low QOL		
				*n*	%	95% CI
Seizure worry	0–100	71.89	(26.87)	34	26.4%	18.8%–34.0%
Overall QOL	0–100	72.02	(16.96)	39	30.2%	22.3%–38.2%
Emotional well‐being	0–100	76.21	(21.10)	34	26.4%	18.8%–34.0%
Energy/Fatigue	0–100	70.74	(19.80)	24	18.6%	11.9%–25.3%
Cognitive	0–100	72.78	(22.30)	36	27.9%	20.2%–35.6%
Medication effects	0–100	69.96	(27.03)	35	27.1%	19.5%–34.8%
Social function	0–100	78.74	(24.35)	33	25.6%	18.1%–33.1%
Overall score	0–100	**74.04**	**(17.22)**	**30**	**23.3%**	**16.0%–30.5%**
VAS score						
Mean (SD)	75.62 (18.02)					
Median (min:max)	80 (0:100)					

*Note:* The bolded values represent the total score of quality of life.

Disparities in QOL were observed when stratifying the PWE by sociodemographic and seizure control. A clear distinction was found based on age, where the elderly PWE group demonstrated a significantly lower proportion of low QOL compared with the adult PWE group. This difference constituted −18.4 percentage points (95% CI: −31.9 to −5.0). Similarly, QOL differed according to seizure control status; PWE with controlled seizures were less likely to report low QOL, showing a difference of −15.8 percentage points (95% CI: −31.1 to −0.6) when compared with those with uncontrolled seizures (Supporting Information 2: Table S2).

A detailed comparison across QOL dimensions highlighted specific differences between these groups. In the comparison between age groups, the proportion of low QOL among elderly PWE was lower than that of adult PWE across all dimensions, with the exception of the social function dimension (FDR adjusted p value = 0.094). Regarding seizure control, PWE with controlled seizures consistently reported better QOL results, specifically showing a lower proportion of low QOL compared with those with uncontrolled seizures in the seizure worry (FDR adjusted p value = 0.042) and social function dimensions (FDR adjusted p value = 0.014) (Supporting Information 2: Table S3).

### 3.4. QOL of Caregivers

The mean EQ‐5D‐5L index score was 0.90 points (95% CI: 0.88–0.92), with 51.8% of caregivers reported experiencing problems related to pain or discomfort, whereas all caregivers reported no problems in the self‐care domain (Table 6).

**Table 6:** Quality of life in caregivers (n = 85).

Domains	Possible score	Mean	(SD)	Problems		
				*n*	%	95% CI
Mobility	1–5	1.33	(0.69)	20	23.5%	14.5%–32.6%
Self‐care	1–5	1.00	(0.00)	0	0.0%	0.0%–0.0%
Usual activities	1–5	1.11	(0.41)	7	8.2%	2.4%–14.1%
Pain/discomfort	1–5	1.69	(0.83)	44	51.8%	41.1%–62.4%
Anxiety/depression	1–5	1.61	(0.80)	39	45.9%	35.3%–56.5%
Index score						
Mean (SD)	0.90 (0.11)					
Median (min:max)	0.94 (0.56:1.00)					
VAS score						
Mean (SD)	84.76 (11.05)					
Median (min:max)	90 (50:100)					

A detailed analysis of individual EQ‐5D‐5L dimensions identified specific disparities in the mobility domain related to age and employment status. The elderly caregivers reported problems in the mobility dimension at a higher proportion than their adult counterparts (FDR‐adjusted p value < 0.001). Employed caregivers reported a lower proportion of problems in this domain compared with unemployed individuals (FDR‐adjusted p value = 0.028) (Supporting Information 3: Table S4).

## 4. Discussion

The mean cost of illness for PWE per outpatient visit in this study was 452.27 PPP‐USD from a societal perspective. Of these costs, 58.0% were direct medical costs. This contrasted with findings from Austria, Germany, Poland, the United States, Sweden, Ethiopia, and India, which reported that indirect costs were the largest component [9–12, 36–38]. The finding can be explained by the fact that over 50% of PWE in our study were unemployed, retired, students, and government officials with paid sick leave, and therefore did not incur productivity losses from clinic visits. Notably, far more substantial lifetime productivity losses resulted from epilepsy‐related unemployment. This occurred because PWE who discontinued work were of working age (with a minimum age of 21 years at the time of job termination), resulting in substantial income losses until retirement (age 60), losses that potentially spanned several decades.

Examining the burden of epilepsy against other complex chronic conditions managed within the Thai healthcare system yields critical insights. Our PWE cost structure, characterized by direct medical costs dominance, resembled that of chronic inflammatory neurological disorders [39], which were driven by costly medications. Crucially, this profile contrasted significantly with the economic burden of another long‐term condition managed within the same system, treatment‐resistant depression [40], in which indirect costs accounted for 87% of the total cost. This divergence suggested that the primary economic burden driver depended on the disease′s functional manifestation. Direct medical costs dominated conditions requiring intensive physical treatment, whereas indirect costs overwhelmed those causing severe cognitive and employment impairment. The indirect cost burden, represented by our finding of substantial long‐term unemployment, was the main route to catastrophic household costs in Thailand. Evidence from other noncommunicable diseases in Thailand confirmed this pattern. For instance, studies on tuberculosis [41] and breast cancer [42] demonstrated that direct nonmedical costs and indirect costs were responsible for the majority of out‐of‐pocket expenses borne by patients. Therefore, Thai policy interventions must move beyond mitigating direct medical costs. Strategic investments must address epilepsy‐related unemployment and ensure rapid access to advanced seizure control, as persistent, uncontrolled disease severity consistently drives the highest long‐term socioeconomic costs across chronic illnesses.

Furthermore, our findings revealed that costs of illness differed significantly by age, employment status, residential distance from the hospital, and seizure control. Lower direct medical costs were observed among seizure‐free PWE, who required fewer medications than those with uncontrolled seizures. Additionally, employed PWE had lower direct medical costs than those without employment, a group comprising unemployed individuals, retirees, students, housewives, and monks. Half of the unemployed PWE group were unable to work due to uncontrolled seizures requiring multiple medications to control the seizures (data not shown). Moreover, PWE aged 60 or older had lower indirect costs because most were not required to bear lost productivity costs. Additionally, PWE who resided outside Khon Kaen Province (where the hospital is located) had higher direct nonmedical and indirect expenses due to transportation costs and lost wages from time off work for hospital appointments. These findings are consistent with a study conducted in Northwest Ethiopia, which demonstrated that age and frequency of seizures were associated with a high cost of illness [11]. Similarly, studies in Australia showed a disparity in direct healthcare costs depending on age [43] and unemployment [10]. Moreover, a German study reported weekly seizures as a cost‐driving factor for direct costs and found that age was associated with significantly lower indirect costs [17]. Our findings indicated that direct nonmedical and indirect costs differed by residential distance, which contrasts with the report from Northwest Ethiopia [11]. Notably, 95.3% of PWE in our study utilized personal or chartered vehicles, thereby increasing direct nonmedical costs.

In this study, the average overall QOLIE‐31 score was 74.04 points (95% CI: 71.04–77.04), and 23.3% of PWE experienced low QOL. Additionally, there was a significant difference in low QOL according to age and seizure control. Specifically, the proportion experiencing low QOL was lower among elderly PWE and PWE with controlled seizures compared with younger PWE and those with uncontrolled seizures. Further examination revealed that 81.3% of elderly PWE were seizure‐free in the last month (data not shown). These findings are similar to previous studies. A study in Morocco found that PWE with no seizure episodes during the last month had higher mean QOLIE‐31 scores compared with PWE with seizures [44]. Similarly, among people with drug‐resistant epilepsy, those with higher seizure frequency had lower mean QOLIE‐31 scores [45].

Caregivers reported a mean EQ‐5D‐5L index score of 0.90 and a mean VAS score of 84.76 points, equivalent to the general Thai population [46]. However, despite these high overall QOL scores, over half of caregivers reported experiencing pain/discomfort (51.8%) and nearly half reported anxiety/depression (45.9%), indicating selective burden in specific health domains. This pattern of high overall scores coupled with prevalent domain‐specific problems has been similarly documented in studies from the United States [47], Georgia [48], and China [49], suggesting that caregiving burden may manifest primarily in psychological and physical dimensions rather than overall QOL. Furthermore, elderly caregivers had a lower mean EQ‐5D‐5L index score than younger caregivers (data not shown). When examining individual domains, mobility problems were more prevalent among older caregivers than younger caregivers. Moreover, unemployed caregivers reported more mobility problems than employed caregivers, which may reflect the age distribution, as over 70% of unemployed caregivers were elderly. These findings align with a previous study examining caregiver burden in adult epilepsy, which found that the caregivers were mainly elderly parents (mean age 66.6 years, SD = 6.7) who experienced physical burden, including health deterioration, excessive workload, fatigue, and sleep disturbance [50].

The primary strength of this study was the use of actual patient‐reported income data rather than population‐average wages. For absenteeism, actual daily income losses were collected from each patient, accounting for occupational differences rather than applying a uniform minimum wage. For unemployment, preunemployment salaries and job loss years were collected, adjusted to 2023 constant prices and discounted to present value. This individualized approach minimized bias from wage averaging and captured the substantial heterogeneity in earnings across occupations, providing more accurate estimates than methods assuming uniform wage rates. Nevertheless, this study had several limitations. First, the reliance on patient self‐reports for retrospective data raises the potential for recall bias. Second, data collection occurred over only 5 months, capturing 129 patients (27.3% of the annual clinic population, or 50.4% of patients attending during this period). However, since patients in our outpatient clinic attend regularly scheduled follow‐up visits for clinical monitoring and medication refills rather than seeking emergency or acute care that might be affected by seasonal variations, healthcare utilization patterns and associated costs remain relatively stable throughout the year. Third, although we employed consecutive sampling by recruiting all eligible patients, data collection was limited to patients who attended clinic appointments in person. Approximately 30% of our regular patients had family members or caregivers collect medications on their behalf, rather than attending in person. These included elderly patients, those with disabilities or socioeconomic barriers preventing clinic attendance, as well as patients with well‐controlled epilepsy utilizing telemedicine services with home medication delivery. These patients were not included in the study. Consequently, our sample could not fully represent the cost spectrum of epilepsy. Exclusion of patients with severe disability or socioeconomic challenges could lead to underestimation of costs, particularly disability‐related expenses (e.g., assistive devices and specialized transportation) and productivity losses. Conversely, exclusion of highly stable patients using telemedicine may lead to overestimation of average healthcare utilization costs. Fourth, as a single‐center study conducted at a tertiary referral hospital, the findings may not be generalizable to all people with epilepsy in Thailand, particularly those receiving care at primary or secondary hospitals or in other geographical regions. Fifth, we lacked details on the severity of epilepsy, which is essential for understanding the cost of illness and QOL for both PWE and caregivers. Furthermore, most PWE who visited our outpatient clinic could control the seizures; therefore, the hospitalizations and laboratory costs comprising direct medical costs were not computed. Sixth, the cross‐sectional design of the study limits our ability to establish definitive causal relationships. While we observed differences between costs and characteristics of patients and caregivers, these findings could not infer causality. Finally, direct medical expenditures in this study were estimated from a societal perspective; however, it is necessary to mention that most patients, such as those in the universal coverage scheme, are eligible for a co‐payment exemption. As a result, the direct medical costs and actual expenses paid by these PWEs were zero. Exploring out‐of‐pocket expenditures from a patient′s perspective presents an important area of study to understand the true economic burden that patients must bear.

## 5. Conclusion

Direct medical costs were the largest component of the total cost of illness in PWE. Costs were significantly lower among PWE who were elderly, employed, and had controlled seizures. Costs increased with greater distance from the hospital. Additionally, epilepsy‐related unemployment resulted in substantial lifetime productivity losses. Overall QOL scores were high for both PWE and caregivers. However, QOL varied by age and seizure control in PWE and by age and unemployment status in caregivers.

## Author Contributions

P.S., S.P., L.W., S.M., and S.T. contributed to the conceptualization and design of the study. P.S. and S.M. managed and controlled the quality of data collection. P.S. performed data analysis and drafted and reviewed the manuscript. S.P. and L.W. interpreted the health economics data. S.T. interpreted the epilepsy data.

## Funding

This study was supported by Young Research Development Project of Khon Kaen University Year 2023.

## Disclosure

All authors read and approved the final manuscript.

## Ethics Statement

This study received approval from the Human Research Ethics Committee of Khon Kaen University, Thailand (Reference Number: HE661028). All participants provided written informed consent prior to participation.

## Conflicts of Interest

The authors declare no conflicts of interest.

## Supporting Information

Additional supporting information can be found online in the Supporting Information section.

## Supporting information


**Supporting Information 1** Sensitivity analysis of lifetime productivity loss due to unemployment.

**Supporting Information 2** Difference in low quality of life in patients with epilepsy.

**Supporting Information 3** Difference in low quality of life in caregivers.

## Data Availability

The data that support the findings of this study are available on request from the corresponding author. The data are not publicly available due to privacy or ethical restrictions.
