# Supplementary material for: The Burden of Epilepsy in Thailand: Estimating Societal Costs and Quality of Life
Source: Behav Neurol. 2026 Mar 5;2026:2885542. doi: 10.1155/bn/2885542 (PMC13580846; doi:10.1155/bn/2885542)
Supplement: Supplementary file 1 [file BN-2026-2885542-s001.docx]

**Table S4A** Domain-specific effects of sex on quality of life in caregivers (n = 85)

| **QOL domain** | **Female (n = 62)** | | **Male (n = 23)** | | **Difference**  **%** | **95%CI** | **p-value** | **FDR-adjusted p-value** |
| --- | --- | --- | --- | --- | --- | --- | --- | --- |
|  | **Problems** | | **Problems** | |  |  |  |  |
|  | **n** | **%** | **n** | **%** |  |  |  |  |
| Mobility | 16 | 25.8 | 4 | 17.4 | 8.4 | -10.5 to 27.4 | 0.384 | 0.877 |
| Usual activities | 5 | 8.1 | 2 | 8.7 | -0.6 | -14.0 to 12.7 | 0.926 | 0.926 |
| Pain/discomfort | 33 | 53.2 | 11 | 47.8 | 5.4 | -18.5 to 29.3 | 0.658 | 0.877 |
| Anxiety/depression | 27 | 43.6 | 12 | 52.2 | -8.6 | -32.5 to 15.2 | 0.479 | 0.877 |

**Table S4B** Domain-specific effects of age on quality of life in caregivers (n = 85)

| **QOL domain** | **Age ≥ 60 (n = 22)** | | **Age < 60 (n = 63)** | | **Difference**  **%** | **95%CI** | **p-value** | **FDR-adjusted p-value** |
| --- | --- | --- | --- | --- | --- | --- | --- | --- |
|  | **Problems** | | **Problems** | |  |  |  |  |
|  | **n** | **%** | **n** | **%** |  |  |  |  |
| Mobility | 13 | 59.1 | 7 | 11.1 | 48.0 | 26.0 to 69.9 | <0.001 | <0.001 |
| Usual activities | 2 | 9.1 | 5 | 7.9 | 1.2 | -12.6 to 14.9 | 0.869 | 0.869 |
| Pain/discomfort | 14 | 63.6 | 30 | 47.6 | 16.0 | -7.6 to 39.6 | 0.183 | 0.366 |
| Anxiety/depression | 12 | 54.6 | 27 | 42.9 | 11.7 | -12.4 to 35.8 | 0.342 | 0.456 |

**Table S4C** Domain-specific effects of employment status on quality of life in caregivers (n = 129)

| **QOL domain** | **Employment (n = 64)** | | **Unemployment (n = 21)** | | **Difference**  **%** | **95%CI** | **p-value** | **FDR-adjusted p-value** |
| --- | --- | --- | --- | --- | --- | --- | --- | --- |
|  | **Problems** | | **Problems** | |  |  |  |  |
|  | **n** | **%** | **n** | **%** |  |  |  |  |
| Mobility | 10 | 15.6 | 10 | 47.6 | -32.0 | -55.1 to -8.9 | 0.007 | 0.028 |
| Usual activities | 5 | 7.8 | 2 | 9.5 | -1.7 | -15.9 to 12.5 | 0.813 | 0.813 |
| Pain/discomfort | 34 | 53.1 | 10 | 47.6 | 5.5 | -19.1 to 30.1 | 0.661 | 0.813 |
| Anxiety/depression | 26 | 40.6 | 13 | 61.9 | -21.3 | -45.3 to 2.7 | 0.082 | 0.164 |

**Table S4D** Domain-specific effects of full-time PWE caring on quality of life in caregivers (n = 129)

| **QOL domain** | **Full-time caring (n = 33)** | | **Part-time caring (n = 52)** | | **Difference**  **%** | **95%CI** | **p-value** | **FDR-adjusted p-value** |
| --- | --- | --- | --- | --- | --- | --- | --- | --- |
|  | **Problems** | | **Problems** | |  |  |  |  |
|  | **n** | **%** | **n** | **%** |  |  |  |  |
| Mobility | 9 | 27.3 | 11 | 21.2 | 6.1 | -12.7 to 24.9 | 0.524 | 0.950 |
| Usual activities | 3 | 9.1 | 4 | 7.7 | 1.4 | -10.8 to 13.6 | 0.822 | 0.950 |
| Pain/discomfort | 19 | 57.6 | 25 | 48.1 | 9.5 | -12.2 to 31.2 | 0.390 | 0.950 |
| Anxiety/depression | 15 | 45.5 | 24 | 46.2 | -0.7 | -22.4 to 21.0 | 0.950 | 0.950 |
