# Supplementary material for: The Burden of Epilepsy in Thailand: Estimating Societal Costs and Quality of Life
Source: Behav Neurol. 2026 Mar 5;2026:2885542. doi: 10.1155/bn/2885542 (PMC13580846; doi:10.1155/bn/2885542)
Supplement: Supplementary file 2 [file BN-2026-2885542-s002.docx]

**Table S2** Difference in low quality of life in patients with epilepsy (n = 129)

| **Characteristics** | **n** | **Mean** | **SD** | **Low QOL** | | **Difference (%)** | **95%CI** | **p-value** |
| --- | --- | --- | --- | --- | --- | --- | --- | --- |
|  |  |  |  | **n** | **%** |  |  |  |
| **Overall score** |  |  |  |  |  |  |  |  |
| Sex |  |  |  |  |  |  |  |  |
| Male | 52 | 74.03 | (17.95) | 15 | 28.9 | Ref. |  |  |
| Female | 77 | 74.04 | (16.83) | 15 | 19.5 | -9.4 | -24.5 to 5.8 | 0.226 |
| Age (years) |  |  |  |  |  |  |  |  |
| < 60 | 97 | 71.46 | (17.54) | 27 | 27.8 | Ref. |  |  |
| ≥ 60 | 32 | 81.85 | (17.73) | 3 | 9.4 | -18.4 | -31.9 to -5.0 | 0.007 |
| Level of education |  |  |  |  |  |  |  |  |
| Basic | 71 | 73.88 | 16.45 | 16 | 22.5 | Ref. |  |  |
| Post-secondary | 58 | 74.23 | 18.27 | 14 | 24.1 | 1.6 | -13.1 to 16.3 | 0.831 |
| Employment status |  |  |  |  |  |  |  |  |
| Unemployed | 58 | 73.16 | 18.09 | 14 | 24.1 | Ref. |  |  |
| Employed | 71 | 74.75 | 16.57 | 16 | 22.5 | -1.6 | -16.3 to 13.1 | 0.831 |
| Seizure-free |  |  |  |  |  |  |  |  |
| No | 52 | 68.52 | (18.48) | 17 | 32.7 | Ref. |  |  |
| Yes | 77 | 77.77 | (15.34) | 13 | 16.9 | -15.8 | -31.1 to -0.6 | 0.042 |

**Table S3A** Domain-specific effects of sex on quality of life (n = 129)

| **QOL domain** | **Female (n = 77)** | | **Male (n = 52)** | | **Difference**  **%** | **95%CI** | **p-value** | **FDR-adjusted p-value** |
| --- | --- | --- | --- | --- | --- | --- | --- | --- |
|  | **Low QOL** | | **Low QOL** | |  |  |  |  |
|  | **n** | **%** | **n** | **%** |  |  |  |  |
| Seizure worry | 21 | 27.3 | 13 | 25.0 | 2.3 | -13.1 to 17.7 | 0.773 | 0.901 |
| Overall QOL | 25 | 32.5 | 14 | 26.9 | 5.6 | -10.4 to 21.5 | 0.496 | 0.901 |
| Emotional well-being | 21 | 27.3 | 13 | 25.0 | 2.3 | -13.1 to 17.7 | 0.773 | 0.901 |
| Energy/Fatigue | 15 | 19.5 | 9 | 17.3 | 2.2 | -11.4 to 15.7 | 0.754 | 0.901 |
| Cognitive | 22 | 28.6 | 14 | 26.9 | 1.7 | -14.1 to 17.4 | 0.837 | 0.901 |
| Medication effects | 24 | 31.2 | 11 | 21.2 | 10.0 | -5.2 to 25.2 | 0.196 | 0.901 |
| Social function | 20 | 26.0 | 13 | 25.0 | 1.0 | -14.3 to 16.3 | 0.901 | 0.901 |

**Table S3B** Domain-specific effects of age on quality of life (n = 129)

| **QOL domain** | **Age ≥ 60 (n = 32)** | | **Age < 60 (n = 97)** | | **Difference**  **%** | **95%CI** | **p-value** | **FDR-adjusted p-value** |
| --- | --- | --- | --- | --- | --- | --- | --- | --- |
|  | **Low QOL** | | **Low QOL** | |  |  |  |  |
|  | **n** | **%** | **n** | **%** |  |  |  |  |
| Seizure worry | 2 | 6.3 | 32 | 33.0 | -26.7 | -39.3 to -14.2 | <0.001 | <0.001 |
| Overall QOL | 4 | 12.5 | 35 | 36.1 | -23.6 | -38.5 to -8.7 | 0.002 | 0.004 |
| Emotional well-being | 3 | 9.4 | 31 | 32.0 | -22.6 | -36.3 to -8.9 | 0.001 | 0.002 |
| Energy/Fatigue | 2 | 6.3 | 22 | 22.7 | -16.4 | -28.3 to -4.6 | 0.006 | 0.008 |
| Cognitive | 5 | 15.6 | 31 | 32.0 | -16.4 | -32.0 to -0.7 | 0.041 | 0.048 |
| Medication effects | 3 | 9.4 | 32 | 33.0 | -23.6 | -37.4 to -9.9 | 0.001 | 0.002 |
| Social function | 5 | 15.6 | 28 | 28.9 | -13.3 | -28.7 to 2.2 | 0.094 | 0.094 |

**Table S3C** Domain-specific effects of education level on quality of life (n = 129)

| **QOL domain** | **Post-secondary (n = 58)** | | **Basic (n = 71)** | | **Difference**  **%** | **95%CI** | **p-value** | **FDR-adjusted p-value** |
| --- | --- | --- | --- | --- | --- | --- | --- | --- |
|  | **Low QOL** | | **Low QOL** | |  |  |  |  |
|  | **n** | **%** | **n** | **%** |  |  |  |  |
| Seizure worry | 17 | 29.3 | 17 | 23.9 | 5.4 | -10.0 to 20.7 | 0.493 | 0.917 |
| Overall QOL | 18 | 31.0 | 21 | 29.6 | 1.4 | -14.5 to 17.4 | 0.858 | 0.917 |
| Emotional well-being | 14 | 24.1 | 20 | 28.2 | -4.1 | -19.2 to 11.2 | 0.603 | 0.917 |
| Energy/Fatigue | 9 | 15.5 | 15 | 21.1 | -5.6 | -18.9 to 7.7 | 0.409 | 0.917 |
| Cognitive | 17 | 29.3 | 19 | 26.8 | 2.5 | -13.0 to 18.1 | 0.749 | 0.917 |
| Medication effects | 16 | 27.6 | 19 | 26.8 | 0.8 | -14.6 to 16.3 | 0.917 | 0.917 |
| Social function | 17 | 29.3 | 16 | 22.5 | 6.8 | -8.4 to 22.0 | 0.383 | 0.917 |

**Table S3D** Domain-specific effects of employment status on quality of life (n = 129)

| **QOL domain** | **Employment (n = 71)** | | **Unemployment (n = 58)** | | **Difference**  **%** | **95%CI** | **p-value** | **FDR-adjusted p-value** |
| --- | --- | --- | --- | --- | --- | --- | --- | --- |
|  | **Low QOL** | | **Low QOL** | |  |  |  |  |
|  | **n** | **%** | **n** | **%** |  |  |  |  |
| Seizure worry | 21 | 29.6 | 13 | 22.4 | 7.2 | -7.9 to 22.3 | 0.352 | 0.749 |
| Overall QOL | 23 | 32.4 | 16 | 27.6 | 4.8 | -11.0 to 20.6 | 0.552 | 0.749 |
| Emotional well-being | 20 | 28.2 | 14 | 24.1 | 4.1 | -11.2 to 19.2 | 0.603 | 0.749 |
| Energy/Fatigue | 14 | 19.7 | 10 | 17.2 | 2.5 | -10.9 to 15.9 | 0.718 | 0.749 |
| Cognitive | 19 | 26.8 | 17 | 29.3 | -2.5 | -18.1 to 13.0 | 0.749 | 0.749 |
| Medication effects | 22 | 31.0 | 13 | 22.4 | 8.6 | -6.6 to 23.8 | 0.269 | 0.749 |
| Social function | 15 | 21.1 | 18 | 31.0 | -9.9 | -25.1 to 5.3 | 0.202 | 0.749 |

**Table S3E** Domain-specific effects of controlled seizure on quality of life (n = 129)

| **QOL domain** | **Controlled seizure**  **(n = 77)** | | **Uncontrolled seizure**  **(n = 52)** | | **Difference**  **%** | **95%CI** | **p-value** | **FDR-adjusted p-value** |
| --- | --- | --- | --- | --- | --- | --- | --- | --- |
|  | **Low QOL** | | **Low QOL** | |  |  |  |  |
|  | **n** | **%** | **n** | **%** |  |  |  |  |
| Seizure worry | 14 | 18.2 | 20 | 38.5 | -20.3 | -36.1 to -4.5 | 0.012 | 0.042 |
| Overall QOL | 18 | 23.4 | 21 | 40.1 | -17.0 | -33.4 to -0.7 | 0.041 | 0.096 |
| Emotional well-being | 17 | 22.1 | 17 | 32.7 | -10.6 | -26.4 to 5.2 | 0.187 | 0.262 |
| Energy/Fatigue | 14 | 18.2 | 10 | 19.2 | -1.0 | -14.8 to 12.7 | 0.881 | 0.881 |
| Cognitive | 17 | 22.1 | 19 | 36.5 | -14.4 | -30.5 to 1.6 | 0.077 | 0.135 |
| Medication effects | 19 | 24.7 | 16 | 30.8 | -6.1 | -21.9 to 9.7 | 0.450 | 0.525 |
| Social function | 14 | 18.2 | 19 | 36.5 | -18.3 | -34.0 to -2.7 | 0.002 | 0.014 |
