# Supplementary material for: The Burden of Epilepsy in Thailand: Estimating Societal Costs and Quality of Life
Source: Behav Neurol. 2026 Mar 5;2026:2885542. doi: 10.1155/bn/2885542 (PMC13580846; doi:10.1155/bn/2885542)
Supplement: Supplementary file 3 [file BN-2026-2885542-s003.docx]

**Table S1** Sensitivity analysis of lifetime productivity loss due to unemployment under varying income loss assumptions (n = 129)

| **Income loss scenario** | **Mean** | **SD** | **95%CI** | **Median** | **IQR** | **95%CI** |
| --- | --- | --- | --- | --- | --- | --- |
| 100% | 14638.37 | 61200.51 | 5169.17 to 26455.61 | 0.00 | 0.00, 0.00 | 0.00 to 0.00 |
| 75% | 10978.78 | 45900.38 | 3714.46 to 20914.92 | 0.00 | 0.00, 0.00 | 0.00 to 0.00 |
| 50% | 7319.18 | 30600.25 | 2476.30 to 13943.28 | 0.00 | 0.00, 0.00 | 0.00 to 0.00 |
| 25% | 3659.59 | 15300.13 | 1238.15 to 6971.64 | 0.00 | 0.00, 0.00 | 0.00 to 0.00 |
